# The structural and optical properties of GaSb/InGaAs type-II quantum dots grown on InP (100) substrate

**DOI:** 10.1186/1556-276X-7-87

**Published:** 2012-01-25

**Authors:** Zhang Shuhui, Wang Lu, Shi Zhenwu, Cui Yanxiang, Tian Haitao, Gao Huaiju, Jia Haiqiang, Wang Wenxin, Chen Hong, Zhao Liancheng

**Affiliations:** 1 School of Materials Science and Engineering, Harbin Institute of Technology, Harbin, 150001, China; 2 National Laboratory for Condensed Matter Physics, Institute of Physics, Chinese Academy of Sciences, Beijing, 100190, China; 3 Engineering Research Center of Solid-State Lighting, School of Electrical Engineering and Automation, Tianjin Polytechnic University, Tianjin, 300160, China

## Abstract

We have investigated the structural and optical properties of type-II GaSb/InGaAs quantum dots [QDs] grown on InP (100) substrate by molecular beam epitaxy. Rectangular-shaped GaSb QDs were well developed and no nanodash-like structures which could be easily found in the InAs/InP QD system were formed. Low-temperature photoluminescence spectra show there are two peaks centered at 0.75eV and 0.76ev. The low-energy peak blueshifted with increasing excitation power is identified as the indirect transition from the InGaAs conduction band to the GaSb hole level (type-II), and the high-energy peak is identified as the direct transition (type-I) of GaSb QDs. This material system shows a promising application on quantum-dot infrared detectors and quantum-dot field-effect transistor.

## Introduction

Quantum-size nanostructure materials have always been the research focus [1-5]. In recent years, staggered lineup type-II quantum-size nanostructures are of great research interest due to their possible application in many novel devices [6,7]. Notably, a large research effort has been focused on the type-II quantum-size nanostructures composed of III and V direct-bandgap semiconductor materials, such as GaSb/GaAs [8-10], InAlAs/InP [11], InP/InGaP [12,13], InP/GaAs [14], GaAsSb/GaAs [15], and InAs/GaSb [16,17]. The reason is that they offer comparatively large bandgap energies and provide a possibility of covering the whole middle and far-infared optical range for photoelectric devices. Among these material systems, GaSb/GaAs quantum dot [QD] is an outstanding representative since its giant valence band offset, characteristic to this system, may result in practical applications for light-emitting devices in the spectral range of 1 to approximately 1.5 μm, such as in ophthalmology, neurology, and endoscopy [18].

Here, we provided another type-II QD material system, GaSb/InGaAs/InP, as another promising building block for optoelectronic and microelectronic applications. Compared to GaSb/GaAs type-II QDs, the bandgap of this system can be adjusted by both the QD-relevant structural characteristics and the In component in a InGaAs matrix. InP substrate is employed instead of GaAs for two reasons: one is that the lattice of InP is matched with the InGaAs buffer layer which has higher electron mobility; the other is that it may make the absorption peak position easily red shifted to 1.3 to approximately 1.55 μm. All these features show a promising application on the quantum-dot infrared detectors [QDIP] and quantum-dot field-effect transistors [QD-FET].

In this work, we investigated the structural and optical properties of type-II GaSb/InGaAs QDs grown on InP (100) substrate using molecular beam epitaxy [MBE].

## Experiments

GaSb/InGaAs type-II QDs were grown on the (100) semi-insulation Fe-doped InP substrate by a V80 MBE system (VG Semicon, East Grinstead, Weat Sussex, UK). The growth mode followed is the Stranski-Krastanow [SK] mode. Firstly, the surface oxides of the InP substrate were desorbed at a substrate temperature of approximately 500°C. A 500-nm In_0.53_Ga_0.47_As buffer layer matched with the InP substrate was then deposited at a growth rate of 5,000Å/h. Four-monolayer [ML] GaSb QDs were deposited with a slow growth rate of 0.12 ML/s. There was a 3-min growth interruption before and after QD growth. Afterwards, a 30-nm In_0.53_Ga_0.47_As capping layer was grown at a rate of 5,000Å/h. A 50-nm In_0.52_Al_0.48_As barrier layer was grown at a rate of 5,300Å/h. Finally, GaSb QDs were grown for the surface morphology measurements. The growth temperature used for the whole growth process was approximately 480°C. In the growth process of the sample, the InGaAs capping layer was doped with Si.

The morphology measurements of the QDs were characterized by a atomic force microscopy [AFM] and a scanning transmission electron microscope [STEM]. The AFM measurements were conducted in a tapping mode in air, and the STEM measurements were obtained using a Tecnai F20 super-twin machine (FEI Co., Hillsboro, OR, USA). The photoluminescence [PL] measurements were performed for the optical properties of the sample at 20 K using the source of a 532-nm line, and the excitation power is changed from 3 mW to 30 mW.

## Results and discussion

**Figure 1:**
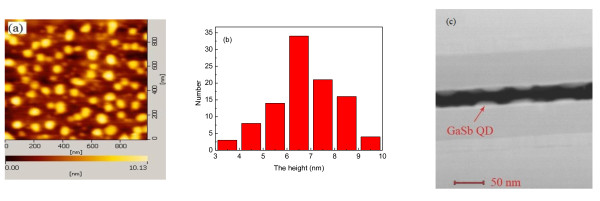
**AFM and STEM images of GaSb/In_0.53_Ga_0.47_As QDs and histogram of the height of GaSb/In_0.53_Ga_0.47_As QDs**. (**a**) The AFM image of GaSb/In_0.53_Ga_0.47_As QDs, (**b**) histogram of the height of GaSb/In_0.53_Ga_0.47_As QDs, and (**c**) the STEM image of GaSb/In_0.53_Ga_0.47_As QDs.

In order to characterize the density, shape, diameter, and height size distribution of GaSb/InGaAs QDs on InP (100) substrate, the AFM and STEM measurements were carried out. Figure 1 shows the AFM and STEM images of GaSb/In_0.53_Ga_0.47_As QDs and the histogram of the height of GaSb/In_0.53_Ga_0.47_As QDs. As shown in Figure 1a, the statistical data indicate that the density of the QDs is approximately 7 × 10^9 ^cm^-2 ^and that the shape of GaSb QDs is rectangular-shaped which is the same with GaSb/GaAs QDs [9]. Figure 1b shows the height distribution of the GaSb/In_0.53_Ga_0.47_As QDs. From the figure, we can see that the height of the quantum dots is mainly concentrated to approximately 6 nm. Due to the well known 'tip effect' of AFM, the results of AFM measurements cannot describe the precise lateral size of the QDs. The STEM measurements were used to image the configuration for overcoming this limitation of AFM measurements. Figure 1c shows that the lateral size of the QDs is approximately 40 nm. The results indicate that the rectangular-shaped GaSb/InGaAs QDs are well developed in the SK growth mode, but no nanodash-like structures which are easily found in the InAs/InP QD system were formed [19]. However, there seemed to be some smaller QDs (the lateral size was about 20 nm) in the AFM image. By measuring the height distribution of the QDs, we observed that they were lower than 2 nm. We did not observe such bimodal distribution in the STEM images. So, we thought that these mound-like structures were possibly from the non-optimized InGaAs buffer layer. Another possible explanation was that the formation of the InGaAsSb wetting layer resulted in the accumulation of individual atoms on the surface to form a mound-like structure, due to the intermixing of As and Sb during the growth of GaSb QD.

**Figure 2:**
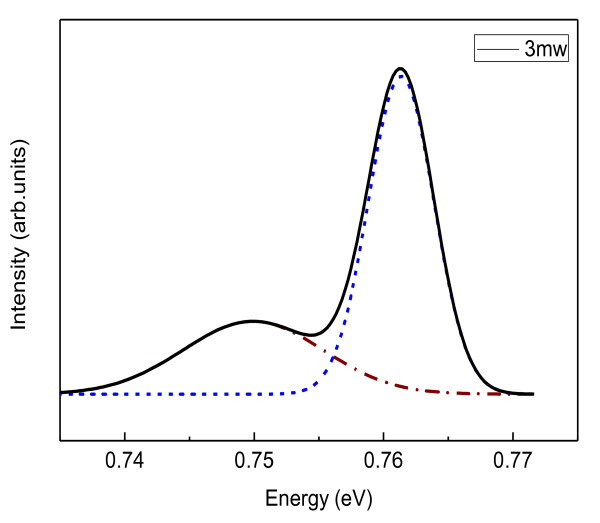
**Low-temperature (20 K) PL spectra of GaSb/InGaAs QD sample on InP substrate**. Dashed-dot and dashed lines show the PL spectra of type-II and type-I, respectively.

**Figure 3:**
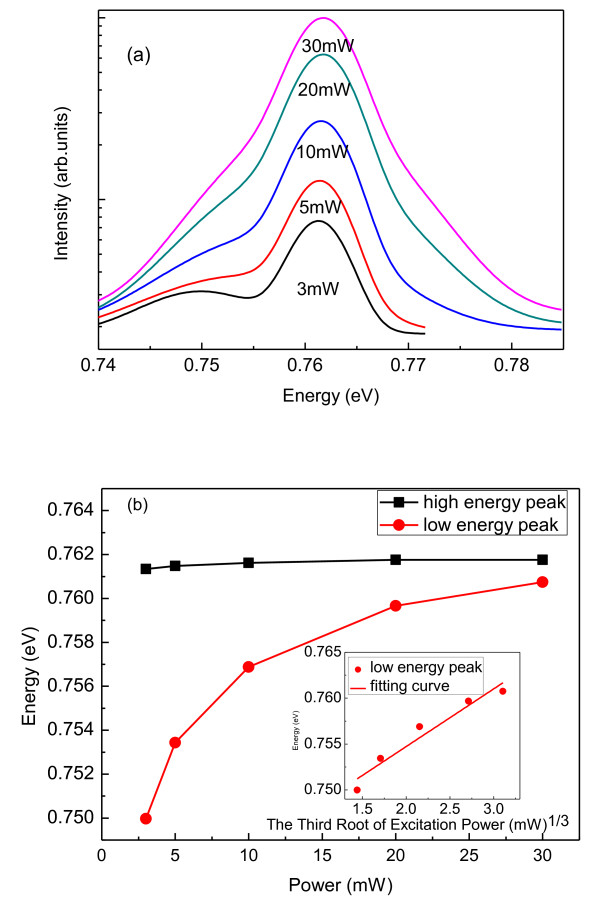
**PL spectra and PL peak energies**. (**a**) The low-temperature PL spectra of the sample measured under different pumping powers from 3 mW to 30 mW; (**b**) The PL peak energies obtained under different excitation powers and the fitting curve of the peak energies with the third root of the excitation power.

**Figure 4:**
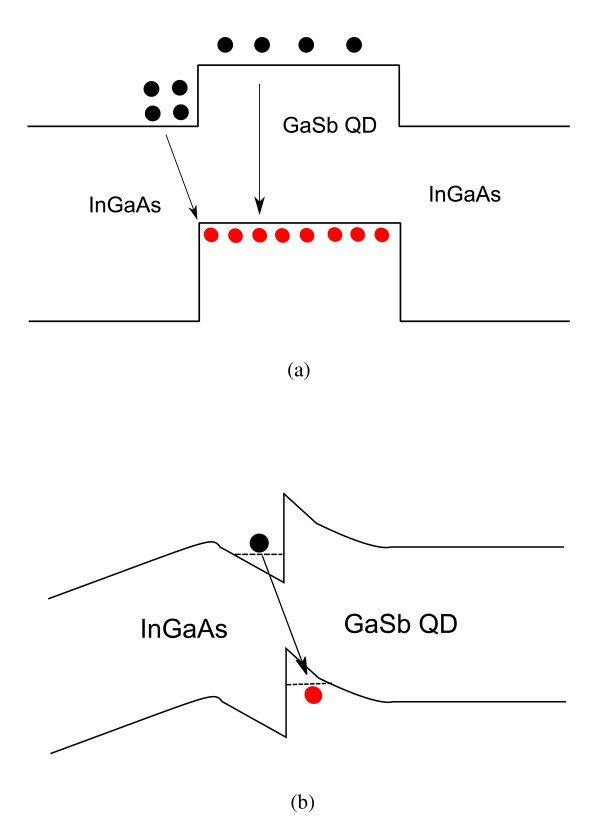
**Schematic band diagrams**. (**a**) Bulk GaSb/InGaAs heterostructures and (**b**) GaSb/InGaAs QD nanostructures forming approximately triangular wells.

Figure 2 shows the PL spectra of four-ML QDs at 20 K with an excitation power of 3 mW. It is obvious that there are two peaks centered at 0.75eV and 0.76eV, respectively. For identifying these two peaks, low-temperature excitation power-dependent PL spectrum tests were carried out, and the results were shown in Figure 3a. Figure 3b shows the PL peak energies with various excitation powers. It is obvious that the low-energy peak blueshifts with the increasing excitation power, while the position of the high-energy peak is almost constant. The PL peak blueshifts with increasing excitation power is a special character of type-II heterostructures. The other supporting evidence of the type-II luminescence is the linear dependence of the PL peak energies over the third root of the excitation density [20]. The inset of Figure 3b shows the linear dependence of the PL peak energies and the third root of the excitation power. Many researchers attributed the high energy PL peak to the transition of the wetting layer [9,10,21]. In these references, there is a common point where the wetting layer peak blueshifts also with increasing excitation power (type-II). However, the high-energy peak in our work is almost independent of the excitation power which is a typical feature of the type-I band transition. Therefore, the interband transition of the GaSb QD would be the only proper origin of the high-energy peak. In the growth process of the sample, the InGaAs cap layer was doped with Si. Because the dope concentration was relatively high, the Fermi level of the InGaAs layer may possibly be higher than the bottom of the conduction band of GaSb QDs. In such circumstance, the light emission intensity of the GaSb QD could be stronger than the type II transition due to the stronger spatial confinement of carriers in the QD and the nature of the direct transition type I transition. It may be the reason that the PL intensity of the direct interband transition (type I) was strong as observed in the experiment. So, these two peaks are identified as the indirect transition from the InGaAs conduction band to the GaSb hole level (type-II) and the GaSb QDs direct interband transition (type-I) respectively, as shown in Figure 4a.

To explain the PL mechanisms of the type-II GaSb/InGaAs QD structures, the schematic band diagrams of GaSb/InGaAs heterostructures are provided in Figure 4b. The spatial separation of electrons and holes will produce an electric field near the type-II GaSb/InGaAs interface. This electric field could make the band bended and form approximately triangular wells adjacent to the heterojunction. With increasing excitation power, the accumulation of electrons and holes at the GaAs/InGaAs interface would steepen the wells. In this case, upraised energy levels in the approximately triangular wells of electrons and holes would cause the PL peak to blueshift.

As is well known, the GaSb QDs only confine the holes, while the electrons are confined in the InGaAs matrix in the type-II GaSb/InGaAs QD heterostructure. We can take advantage of these features to accomplish a charge-discharge process of QDs and then to modulate the electric property of a two-dimensional electron gas [2DEG] in QD-FET. In this kind of QD-FET structure, type-II GaSb/InGaAs QDs are embedded; even if the GaSb QDs directly contacts with the 2DEG, electrons will still be blocked by the GaSb barrier and will not enter the QDs. Therefore, this structure will prolong the lifetime of the holes. So, the QD-FET based on the above band structure can be used to improve the sensitivity of existing InAs/GaAs QD-FET. In addition, this material system can be fabricated on InP substrates. The higher electron mobility InGaAs light absorption layer with lattice matched to the InP substrate has a strong optical absorption in the range of 1.3 to approximately 1.55 μm which is the low-loss optical fiber window. All of these features will promote the application of QD-FET on quantum communications, night vision, and other fields. Besides, owing to the spatially separated electrons and hole characters of type-II QDs, the GaSb/InGaAs QD-based QDIP could have obviously better performance than the InAs/(In)GaAs QD-based QDIP.

## Conclusion

We have investigated the structural and optical properties of self-organized type-II GaSb/InGaAs heterostructure QDs grown on InP (100) using MBE. Formation of type-II GaSb/InGaAs heterostructure QDs centered on the PL peak at 0.75eV at 20 K. This type-II luminescence originates from radiative recombination of spatially separated electrons and holes. The PL peak positions are in proportion to the third root of the excitation power, which is a direct evidence of type-II luminescence. This structure was proposed for many important applications such as tunable laser, quantum-dot infrared detectors, and QD-FET.

## Competing interests

The authors declare that they have no competing interests.

## Authors' contributions

ZS participated in the MBE growth, carried out the PL measurements, and drafted the manuscript. CY conducted the STEM measurement. SZ, TH, and GH conducted the MBE growth. JH, WW, and CH coordinated the study. WL provided the idea and conceived the study together with ZL. All authors read and approved the final manuscript.
